# Supplementary material for: Evaporation of microwave-shielded polar molecules to quantum degeneracy
Source: Nature. 2022 Jul 27;607(7920):677–81. doi: 10.1038/s41586-022-04900-0 (PMC9329123; doi:10.1038/s41586-022-04900-0)
Supplement: Supplementary file 1 — This file contains Supplementary Methods comprising the characterization of the microwave Rabi frequency, the characterization of the microwave polarization, the microwave transitions used for these characterizations and the coupled-channel calculations. Included are Supplementary Figs. 1 and 2 with legends. [file 41586_2022_4900_MOESM1_ESM.pdf]

nature portfolio

https://doi.org/10.1038/s41586-022-04900-0

## Supplementary information

# Evaporation of microwave-shielded polar molecules to quantum degeneracy

In the format provided by the authors and unedited

# Supplementary Information for
# Evaporation of microwave-shielded polar molecules to quantum degeneracy

Andreas Schindewolf,[1,2] Roman Bause,[1,2] Xing-Yan Chen,[1,2] Marcel Duda,[1,2] Tijs Karman,[3] Immanuel Bloch,[1,2,4] and Xin-Yu Luo[1,2]

[1] *Max-Planck-Institut für Quantenoptik, 85748 Garching, Germany*
[2] *Munich Center for Quantum Science and Technology, 80799 München, Germany*
[3] *Institute for Molecules and Materials, Radboud University, Heijendaalseweg 135, 6525 AJ Nijmegen, Netherlands*
[4] *Fakultät für Physik, Ludwig-Maximilians-Universität, 80799 München, Germany*

### Microwave transitions

Figure 1 of the main text shows only the most essential parts of the energy level structure. The full hyperfine structure of the excited rotational state $J = 1$ is presented in Supplementary Fig. 1a. In contrast to the situation in CaF [51], NaK has no fine structure in the electronic ground state. Consequently the rotationally excited states that can couple to the absolute ground state, which have $(m_{i,\mathrm{Na}}, m_{i,\mathrm{K}}) = (3/2, -4)$ character, are spread over just a few hundred kilohertz, much less than the microwave detunings used for shielding. Here, $m_{i,\mathrm{Na}}$ and $m_{i,\mathrm{K}}$ are the projections of the nuclear spins of Na and K onto the magnetic field axis, respectively. At 72.35 G a $\sigma^-$-polarized microwave couples mainly to the $|J = 1, m_J = -1\rangle$ state with transition frequency 5.643 4137 GHz. In absence of electric fields, the transition dipole moment (TDM) is already $0.96\, d_0/\sqrt{3}$. In a strong microwave field, the nuclear spin projections are further purified bringing the TDM even closer to the maximum value of $d_0/\sqrt{3}$.

Unfortunately, we cannot directly measure the coupling strength $\Omega$ at full microwave power by driving resonant Rabi oscillations. Coupling to weaker transitions would lead to beat signals with the main Rabi oscillations. However, we can measure the effective Rabi frequency $\Omega_{\mathrm{eff}}$ at detunings that are large enough to suppress coupling to unwanted transitions. For these measurements we temporarily switch off the optical dipole traps to avoid ac Stark shifts from the trapping light. We then generate a rectangular microwave pulse with a fast microwave switch that controls the input of our microwave amplifier. The microwave pulse drives Rabi oscillations between the rotational states, as shown in Supplementary Fig. 1b. We ignore the oscillations during the first 5 µs of the microwave pulse, as the amplifier requires some time to reach full output power. These measurements are performed at low molecule density in order to suppress dephasing and inelastic collisions between the molecules. From the measurements of $\Omega_{\mathrm{eff}}$ shown in Supplementary Fig. 1c we deduce $\Omega \approx 2\pi \times 11$ MHz.

### Microwave polarization

It is crucial that the microwave field has a high degree of polarization purity in order to achieve efficient shielding. Especially for small detunings, unwanted polarization components can couple to excited states with different $m_J$ character which significantly reduces the shielding effect [52]. Fortunately, the strong ac electric field of the microwave redefines the quantization axis of the molecules so that we only have to consider $\sigma^+$ and $\sigma^-$ components in the microwave frame. We can use different microwave transitions of the molecules to characterize the polarization of the microwave field in situ. However, in order to resolve the individual transitions, only weak microwave fields can be applied, i.e., the microwave polarization can only be characterized in the frame of the dc magnetic field. We probe the microwave field polarization at 135 G, where we can still stabilize the dc magnetic field and where the used transitions, marked in Supplementary Fig. 1a, are reasonably isolated. The measurements, shown in Supplementary Fig. 1(d–f), are performed similarly to the measurements of $\Omega_{\mathrm{eff}}$, described earlier. However, here we measure on resonance and the microwave power is attenuated by 55–61 dB. The microwave power has to be low enough to avoid off-resonant coupling to neighbouring transitions but strong enough to realize Rabi oscillations of at least $2\pi \times 2$ kHz, because we can only turn off the dipole traps for about 1 ms before we start losing molecules. The TDMs of the selected $\sigma^+$, $\pi$, and $\sigma^-$ transitions are $0.875\, d_0/\sqrt{3}$, $0.789\, d_0/\sqrt{3}$, and $0.989\, d_0/\sqrt{3}$, respectively. From the measured Rabi frequencies, the relative microwave power, and the TDMs, we can determine the ratio of the electric field amplitudes $E_{\sigma^+}/E_{\sigma^-} = 0.169(8)$ and $E_\pi/E_{\sigma^-} = 0.462(30)$. Although we do not know the phase relation between the measured ac electric field components in the frame of the dc magnetic field, we can deduce that the wave vector of the microwave is tilted somewhere between $21.5(12)^\circ$ and $29.0(16)^\circ$ with respect to the magnetic field axis. In the microwave frame the ellipticity angle is then given by the electric field amplitudes $E'_{\sigma^+}$ and $E'_{\sigma^-}$ as $\xi = \arctan\left(E'_{\sigma^+}/E'_{\sigma^-}\right)$ and has a value between $11.5(5)^\circ$ and $5.9(6)^\circ$. To calculate the potential curves in Fig. 1 of the main text and the rate co-

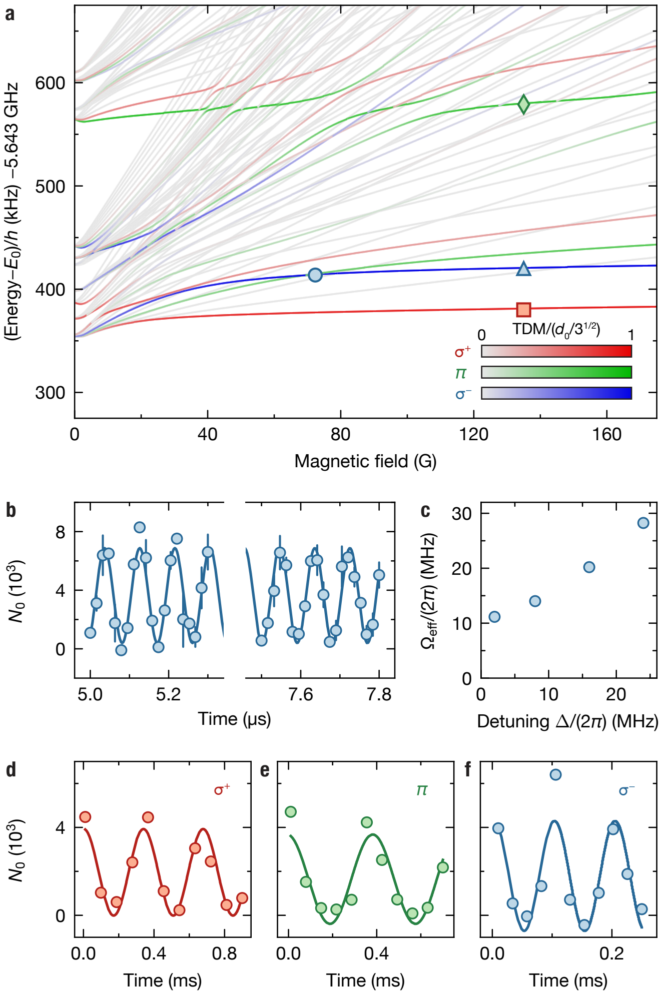


Supplementary Fig. 1. **Microwave transitions. a**, Zeeman diagram of the microwave transition frequencies from the absolute ground state with energy $E_0$ to the hyperfine manifold of the exited rotational state ($J = 1$). The color scales show the transition dipole moment (TDM) for the different microwave polarizations, assuming that the ground state has the nuclear spin projections $(m_{i,\mathrm{Na}}, m_{i,\mathrm{K}}) = (3/2, -4)$, which is a good approximation for magnetic fields larger than 3 G. The state energies and TDMs are calculated with the code from Ref. [49] and the parameters from Ref. [50]. The circle marks the $\sigma^-$ transition that is used for microwave shielding. The diamond, triangle, and square mark the microwave transitions that are used to probe the polarization of the microwave field. **b**, Example for off-resonant Rabi oscillations at full microwave power with a detuning of $2\pi \times 2$ MHz from the $\sigma^-$ transition at 72.35 G. The data show the number of molecules in the absolute ground state $N_0$ and the error bars are the standard error of the mean of two repetitions. The line is a sinusoidal fit function that yields an effective Rabi frequency $2\pi \times 11.157(6)$ MHz. **c**, Effective Rabi frequency $\Omega_{\mathrm{eff}}$ at full microwave power versus detuning from the $\sigma^-$ transition at 72.35 G. **d**–**f**, Examples of resonant Rabi oscillations at the $\sigma^+$ (**d**), $\pi$ (**e**), and $\sigma^-$ (**f**) transitions indicated in **a** at 135 G. The sinusoidal fit functions yield Rabi frequencies of $2\pi \times 2.96(8)$ kHz, $2\pi \times 2.58(13)$ kHz, and $2\pi \times 9.9(4)$ kHz, respectively. The power of the microwave is attenuated by 55 dB (**d**) or 61 dB (**e** and **f**).

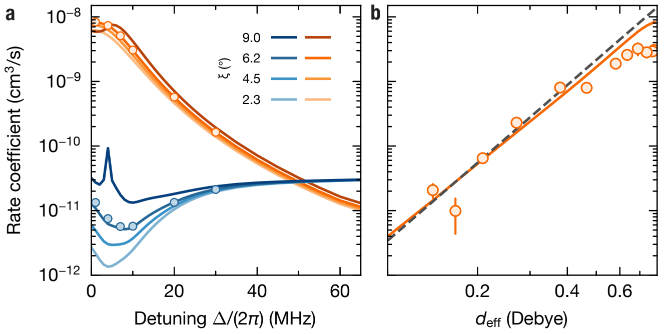


Supplementary Fig. 2. **Scaling of the collision rates. a**, Calculations of the elastic (orange) and inelastic (blue) collision rate coefficients for various microwave ellipticity angles $\xi$. Further simulation parameters are $T = 800$ nK and $\Omega = 2\pi \times 11$ MHz. The markers are calculations including hyperfine interactions for $\xi = 6.2°$. **b**, The circles and the solid line are the measured values and the calculations of the elastic rate coefficients, as shown in Fig. 2 of the main text for $T = 800$ nK. The dashed line is a simplified calculation of the elastic scattering rate based on the effective dipole moment $d_{\mathrm{eff}}$. The error bars are the standard deviation from the fit to the differential equations.

efficients in Fig. 2a of the main text we assume $\xi = 6.2°$.

The ellipticity of the microwave polarization has a significant effect on the inelastic collision rate and therefore on the shielding efficiency, as illustrated in Supplementary Fig. 2a. For large enough ellipticity even inelastic scattering resonances can arise when the undesired circular-polarization component contributes to the coupling at small detunings. In our setup the purity of the circular polarization can partially be optimized by moving and rotating a cylindrical metal sheet that surrounds the helical antenna.

## Coupled-channels calculations

We perform coupled-channels scattering calculations using the framework developed in Refs. [52–54] and here we summarize numerical details of these calculations.

The Hamiltonian describes the NaK molecules as rigid rotors with electric dipole moments that interact with one another as well as with the microwave electric field. Furthermore, the molecules have nuclear spins that couple with one another and with a static magnetic field. The channel basis was truncated by including only the lowest two rotational states $J = 0, 1$ and partial waves $L = 1, 3, 5$. We propagated the scattering wavefunctions from $R_{\mathrm{min}} = 30\,a_0$ to $R_{\mathrm{max}} = 60{,}000\,a_0$, imposing a capture boundary condition at $R_{\mathrm{min}}$ and the usual scattering boundary conditions at $R_{\mathrm{max}}$, from which the $S$-matrix and collision cross sections are obtained. This short-range boundary results in loss rates given by the universal loss model [55] in the absence of external

fields, which is in reasonable but not perfect agreement with experimental loss rates, $4.9 \times 10^{-11}\,\mathrm{cm}^3/\mathrm{s}$ versus $7.7(5) \times 10^{-11}\,\mathrm{cm}^3/\mathrm{s}$ at $T = 800\,\mathrm{nK}$, respectively. We performed scattering calculations for nine values of the collision energy spaced logarithmically between $0.1\,k_\mathrm{B}T$ and $10\,k_\mathrm{B}T$, and subsequently cross sections are multiplied by the velocity and averaged over the Maxwell–Boltzmann or Fermi–Dirac distribution to obtain collision rates compared to experiment in Fig. 2 of the main text.

Scattering rates presented in Fig. 2 of the main text were obtained neglecting hyperfine interactions, and using the microwave polarization determined from the experiment, which is elliptical and tilted with respect to the magnetic field. We have also performed calculations including hyperfine interactions, but their effect is small, as can be seen in Supplementary Fig. 2a. Here we truncated the hyperfine basis by including functions with $\Delta m_i = \pm 1$ only, i.e., functions that differed at most one quantum from the initial state. Convergence tests with $\Delta m_i = \pm 2$ were also performed. This figure also contains scattering rates for microwave polarizations of varying ellipticity. In this case, the polarization lies in the plane perpendicular to the magnetic field axis.

Potential energy curves shown in Fig. 1 of the main text were obtained by diagonalizing the Hamiltonian excluding kinetic energy for fixed $\theta$, the angle between the direction of approach of the colliding molecules and the microwave propagation direction, and for fixed $R$, the distance between the two molecules, as described in Ref. [52]. This omits the centrifugal kinetic energy, which is not well defined for fixed $\theta$, and it has neglected hyperfine interactions for clarity of Fig. 1 of the main text.

The elastic cross section can, to a reasonable degree, be approximated by $(32\pi/15)(\mu d_\mathrm{eff}^2/(\hbar^2 4\pi\varepsilon_0))^2$ with the reduced mass $\mu = m/2$ [56]. A comparison with our coupled-channel calculation is shown in Supplementary Fig. 2b.


[49] Blackmore, J. A., Gregory, P. D., Hutson, J. M. & Cornish, S. L. Diatomic-py: A python module for calculating the rotational and hyperfine structure of $^1\Sigma$ molecules. Preprint at https://arXiv.org/abs/2205.05686 (2022).

[50] Will, S. A., Park, J. W., Yan, Z. Z., Loh, H. & Zwierlein, M. W. Coherent microwave control of ultracold $^{23}$Na$^{40}$K molecules. *Phys. Rev. Lett.* **116**, 225306 (2016).

[51] Anderegg, L. *et al.* Observation of microwave shielding of ultracold molecules. *Science* **373**, 779–782 (2021).

[52] Karman, T. & Hutson, J. M. Microwave shielding of ultracold polar molecules with imperfectly circular polarization. *Phys. Rev. A* **100**, 052704 (2019).

[53] Karman, T. & Hutson, J. M. Microwave shielding of ultracold polar molecules. *Phys. Rev. Lett.* **121**, 163401 (2018).

[54] Karman, T. Microwave shielding with far-from-circular polarization. *Phys. Rev. A* **101**, 042702 (2020).

[55] Idziaszek, Z. & Julienne, P. S. Universal rate constants for reactive collisions of ultracold molecules. *Phys. Rev. Lett.* **104**, 113202 (2010).

[56] Bohn, J. L., Cavagnero, M. & Ticknor, C. Quasi-universal dipolar scattering in cold and ultracold gases. *New J. Phys.* **11**, 055039 (2009).
